# Assessing self-regulation strategies: development and validation of the tempest self-regulation questionnaire for eating (TESQ-E) in adolescents

**DOI:** 10.1186/s12966-014-0106-z

**Published:** 2014-09-02

**Authors:** Emely De Vet, Denise De Ridder, Marijn Stok, Karen Brunso, Adriana Baban, Tania Gaspar

**Affiliations:** Clinical and Health Psychology, Utrecht University, Heidelberglaan 1, 3508 TC Utrecht, The Netherlands; Chair group Strategic Communication, Wageningen University, Hollandseweg 1, 6706 KN Wageningen, The Netherlands; Department of Business Administration, Aarhus University, Bartholins Allé 10, 8000 Aarhus, Denmark; Department of Psychology, Babes-Bolyai University, 37 Republicii, 400015 Cluj-Napoca, Romania; Faculty of Human Kinetics, Technical University of Lisbon, Estrada da Costa1495 - 688 Cruz Quebrada, Lisbon, Portugal

**Keywords:** Self-regulation, Temptations, Goals, Eating, Adolescence, Measurement, Questionnaire, Diet

## Abstract

**Background:**

Applying self-regulation strategies have proven important in eating behaviors, but it remains subject to investigation what strategies adolescents report to use to ensure healthy eating, and adequate measures are lacking. Therefore, we developed and validated a self-regulation questionnaire applied to eating (TESQ-E) for adolescents.

**Methods:**

Study 1 reports a four-step approach to develop the TESQ-E questionnaire (n = 1097). Study 2 was a cross-sectional survey among adolescents from nine European countries (n = 11,392) that assessed the TESQ-E, eating-related behaviors, dietary intake and background characteristics. In study 3, the TESQ-E was administered twice within four weeks to evaluate test-retest reliability (n = 140). Study 4 was a cross-sectional survey (n = 93) that assessed the TESQ-E and related psychological constructs (e.g., motivation, autonomy, self-control). All participants were aged between 10 and 17 years.

**Results:**

Study 1 resulted in a 24-item questionnaire assessing adolescent-reported use of six specific strategies for healthy eating that represent three general self-regulation approaches. Study 2 showed that the easy-to-administer theory-based TESQ-E has a clear factor structure and good subscale reliabilities. The questionnaire was related to eating-related behaviors and dietary intake, indicating predictive validity. Study 3 showed good test-retest reliabilities for the TESQ-E. Study 4 indicated that TESQ-E was related to but also distinguishable from general self-regulation and motivation measures.

**Conclusions:**

The TESQ-E provides a reliable and valid measure to assess six theory-based self-regulation strategies that adolescents may use to ensure their healthy eating.

## Background

The employment of self-regulation strategies can help adolescents to successfully adopt a healthier eating pattern [1–3]. Although many adolescents are aware of the benefits of a healthy diet [4], their food choices show considerable room for improvement [5–7]. For the first time in their lives, adolescents themselves become responsible for (part of) their food choices and despite good intentions to adopt a healthy diet, peer pressure and a desire for autonomy typically challenge these good intentions and may make adolescents more willing to indulge in unhealthy foods [8–10]. Notwithstanding their inclination to engage in unhealthy food practices, adolescents seem to possess the skills required for self-regulation [11–13]. Many of the capacities involved in self-regulation are acquired and developed in adolescence [11] and growth of the prefrontal cortex during the teenage years increasingly enables adolescents to regulate their short-term actions and emotions in the context of long-term goals [12,13]. Self-regulation becomes more focused, efficient and intentional as it evolves to encompass more elaborate long-term planning and goal setting [14], making adolescence an interesting case for studying self-regulation strategies: in theory the capacity for self-regulation is there, but environmental factors may hinder using these strategies. Importantly, when healthy eating patterns are established during this period (by making use of self-regulation), this has been shown to be a good precursor of healthy eating patterns throughout life [15,16]. Learning more about the strategies adolescents report to employ in self-regulating their eating behavior is thus of substantial importance [17], but reliable and comprehensive measures are lacking. The present research aims to fill this gap by 1) identifying strategies adolescents report to use to regulate their eating behavior, and 2) developing and validating a questionnaire to assess the self-reported use of self-regulation strategies for healthy eating in adolescents.

### Defining self-regulation strategies

The term self-regulation is often used to refer broadly to efforts by humans to alter their thoughts, feelings, desires, and actions in the perspective of their personally valued long-term goals [18,19]. For example, self-regulation is required when individuals feel tempted to eat a delicious food, which may interfere with their long-term goal of maintaining a healthy weight. When experiencing such a conflict, people may make small changes to their surroundings, such as choosing to keep tempting foods out of reach [20,21]; plan to substitute an unhealthy food item with a healthy one if they encounter a situation that might lure them into healthy snacking [22,23]; or even make their diet goal salient when confronted with a food temptation [24,25].

Self-regulation theories state that people engage in self-regulation efforts when they experience a discrepancy between their current state and a desired state [18] or when they experience a conflict between an urge for immediate gratification and adherence to a long-term goal [19]. This view of self-regulation suggests that strategies may operate at both sides of the conflict to support goal striving. Strategies may aim at making temptations (or any other ‘short-term goal’ relating to distractions and frustrations) less important or relevant, thereby decreasing the chance that the temptation interferes with efforts for goal pursuit. Alternatively, strategies can aim at making desired states or long-term goals more important or relevant, thereby directly contributing to goal pursuit. A general theoretical and widely accepted categorization of self-regulation strategies indeed distinguishes between ‘cooling down’ temptations (for example by thinking of temptations in an abstract way) and ‘heating up’ goals (for example by contemplating the importance of a goal) [26–28].

A more comprehensive specification of this distinction of temptation-focused versus goal-focused self-regulation proposes that both types can be further broken down into either behavioral action (towards the temptation or the goal) or alteration of the psychological meaning (of the temptation or the goal) - thus proposing four general self-regulation *approaches* (see also Table 1): i) taking action toward a temptation; ii) altering the psychological meaning of a temptation; iii) taking action toward a goal; and iv) altering the psychological meaning of a goal [29]. In turn, each of these four general approaches may take different specific forms. For example, when people want to take action toward a temptation, they may attempt to avoid this temptation by not going into the supermarket when hungry. When people want to change the meaning of a food temptation, they may distract themselves by calling a friend. Alternatively, if they would want to engage in action towards their goal of a healthy diet, they could set a clear rule for themselves, such as only being allowed one piece of candy per day. Finally, if they want to change the meaning of their healthy eating goal, they could reappraise it by deciding that to them, healthy eating no longer means only eating some fruits, but also cutting down on snacks and soft drinks.

**Table 1:** **A theoretical breakdown of self-regulation into four general self-regulation approaches with an example strategy for each approach**

	**Cooling down temptations**	**Heating up goals**
**Behavioral action**	*approach:* action toward a temptation	*approach:* action toward a goal
	*typical strategy:* avoidance of temptations	*typical strategy:* goal and rule setting
**Altering the psychological meaning**	*approach:* altering the psychological meaning of a temptation	*approach:* altering the psychological meaning of a goal
	*typical strategy:* distraction	*typical strategy:* focus on attractiveness of goal

In order to avoid any misunderstanding about the level of abstraction we are referring to, we will adopt the following terms. When we talk about types of self-regulation that are informed by the theoretical distinction between temptation-directed and goal-directed behavioral action or altering the psychological meaning, we will employ the term self-regulation *approaches*. When we talk about specific actions that can be rubricated under one of the four approaches, we use the term *strategies*.

### Empirical identification and assessment of self-regulation strategies

Despite theorizing on self-regulation, this is currently not fully translated to empirical research on healthy eating by adolescents. First, there is currently a lack of consensus on the specific strategies used by adolescents to ensure healthy eating. Studies in adults reveal quite diverse compilations of self-regulation strategies for healthy eating [30,31]. For the regulation of eating behavior, the term self-regulation has been used to cover competencies as broad as ‘the skills necessary to achieve personal goals’ [32] but also to cover an enumeration of strategies as specific as ‘self-instruction, self-observation, self-evaluation, self-reward, problem-solving, and coping’ [31–35]. The most extensive list of strategies involved in self-regulation of healthy eating has been reported in a systematic review of behavior change techniques, proposing 26 strategies related to acting upon healthy eating goals [36]. Although previous research has documented a variety of strategies that adults may employ in self-regulating their eating behavior, attempts to document the strategies that adolescents use to ensure their healthy eating are scarce. Only one study aimed to investigate adolescents eating self-regulation [9], but this study describes so-called self-regulation *cognitions* rather than actual strategies that can be employed to improve self-regulation performance.

Second, valid instruments to assess self-regulation strategies for healthy eating are currently lacking. Available instruments for general self-regulation are not easily adaptable to eating behavior, do not consider specific strategies or have not been examined in adolescents [37,38]. We argue that lack of consensus about the core strategies adolescents use to regulate eating behavior and lack of valid instruments hamper progress in self-regulation research. We therefore developed and validated a measurement instrument of self-regulation strategies that is strongly informed by basic self-regulation theory.

### The present study

The present research aims 1) to identify self-generated self-regulation strategies for healthy eating within the proposed theoretical framework in a large and varied sample of European adolescents and 2) to develop and validate a questionnaire to assess these strategies. Study 1 describes the development of the Tempest Self Regulation Questionnaire for Eating (TESQ-E). In study 2 we present psychometric properties and validity of the questionnaire. Test-retest reliability is described in study 3 and construct validity in study 4.

## Study 1 Development of the TESQ-E

The TESQ-E was developed in an iterative process of four steps, combining bottom-up and top-down processes.

First, a literature review was conducted in three different areas of psychological research that are important to self-regulation of eating behavior in adolescents (health psychology, consumer psychology, and developmental psychology), the main findings of which are reported above. Existing scales for assessing self-regulation strategies were reviewed for their suitability and applicability to eating behavior in adolescents (internal Tempest report)^a^. Briefly repeating the most important finding, the review showed that no valid questionnaire existed to assess the self-regulation strategies for healthy eating among adolescents, and that the development of a new questionnaire to fill this void was thus warranted.

Second, to generate items for the development of a new scale, a two-step qualitative procedure was employed in four different European countries reflecting Northern (Denmark), Western (The Netherlands), Southern (Portugal) and Eastern (Romania) parts of Europe (See [17] for a detailed description of the methodology). This procedure allowed for the collection of statements about self-regulation strategies that adolescents themselves consider relevant for their own healthy eating. In the first step, a sample of 336 adolescents was instructed to complete the sentence “Things I can do myself to ensure my healthy eating, are…” (See Table 2 for an overview of the samples of different studies).

**Table 2:** **Description of different study samples**

		**BE**	**DK**	**FI**	**GE**	**NL**	**PO**	**PT**	**RO**	**UK**	**Total**
**Study 1**											
Step 1											
Step 2	n		77			62		100	97		336
	%		22.9%			18.5%		29.8%	28.8%		100%
	Age range		12-17			12-17		12-17	12-17		12-17
Step 3											
Step 4	n		183			216		176	186		761
	%		24.0%			28.4%		23.1%	24.4%		100%
	Age range		11-17			10-16		10-17	11-19		10-19
**Study 2**											
	n	1134	1165	1157	1397	1274	1445	1189	1401	1230	11392
	%	10.0%	10.2%	10.2%	12.3%	11.2%	12.7%	10.4%	12.3%	10.8%	100%
	Age range	10-17	10-17	10-16	10-17	10-17	10-17	10-17	10-17	10-17	10-17
**Study 3**											
	n								140		140
	%								100%		100%
	Age range								10-17		10-17
**Study 4**											
	n					93					93
	%					100%					100%
	Age range					14-16					14-16

Note. BE = Belgium, DK = Denmark, FI = Finland, GE = Germany, NL = Netherlands, PL = Poland, PO = Portugal, RO = Romania, UK = United Kingdom.

The first step resulted in a collection of 326 statements. In the second step, a Q-sort task was used to determine which of these statements adolescents believe to be most instrumental for ensuring their healthy eating. Additionally, adolescents sorted out statements that they thought belonged together. This was also done by the researchers. The results of both sortings were compared and disagreement was solved with discussion.

Third, the remaining statements after grouping and removing duplicates from the different countries were used to inform the scale. In order to accomplish this, we first transformed statements into items with the format “If I find myself in situation X, I typically do Y” to promote unbiased responses to that particular item. Items were then grouped under strategies, which were informed by labels provided by the participants in the study. Next, strategies were assigned to approaches by using the theoretical framework of self-regulation proposed by Fishbach and Converse [29]. As outlined in the introduction, four self-regulation approaches can be defined (i.e., action towards goals, action towards temptations, change the meaning of the goal and change the meaning of the temptation). Three researchers (EdV, DdR, MS) independently assigned items generated by adolescents in the two-step qualitative procedure to specific strategies and specific strategies to one of four general self-regulation approaches. When assigning the self-generated items to strategies, it appeared that adolescents did not mention behaviors that addressed the psychological meaning of the goal. Therefore, only three self-regulation approaches were included. Assigning the items resulted in six separate self-regulation strategies that can be grouped into three general self-regulation approaches which form one overarching concept of self-regulation. More specifically, the three general approaches each represent two prototypical self-regulation strategies (see also Table 1):

1. Strategies directly addressing temptations:
  - *Avoidance of temptations*: Avoidance of situations that tempt adolescents to buy or consume unhealthy products
  - *Controlling temptations*: Controlling the food environment in such a way that healthy products become easily accessible, and unhealthy products become less accessible
2. Strategies addressing the meaning of temptations:
  - *Distraction:* Re-allocating attention from a tempting food cue to a distraction object or event
  - *Suppression:* Deliberately trying to reduce the impact of tempting food cues on thought, emotions, impulses
3. Strategies directly addressing the goal:
  - *Goal and rule setting*: express explicit intentions, plans or goals to eat healthily
  - *Goal deliberation*: exercises that help to keep the goal focal such as elaborating on consequences of failing or self-monitoring

Fourth and finally, 761 adolescents from four countries (Denmark, The Netherlands, Portugal, and Romania) were presented with an 80-item pilot-version of the TESQ-E scale consisting of the six self-regulation strategies conform the theoretical framework outlined above. We chose to start with an extensive list of items so that the best possible items could be selected. Adolescents were asked to rate to what extent the statements about dealing with food applied to them. They were instructed to think about their behavior in the past two weeks and respond to the items on a 7-point Likert scale (1 = never, 7 = always). In addition, they were invited to highlight items that they considered difficult, vague, or otherwise hard to complete. For all items, Kurtosis, the number of missing values and exploratory factor analyses were computed. Based on the results of step 4, the final TESQ-E with 24 items measuring six separate self-regulation strategies each with four items was constructed (See Table 3 for the full scale and instructions). We chose to include only four items per strategy, because we strived for a concise measure. Only items that none of the participants had identified as difficult to complete, had few missing values, and had a normal Kurtosis (<3) in step four were considered for inclusion. In case more than four items were considered suitable, the four items with the highest factor loadings were selected. Additionally, the answering format of the scale was reduced to a 5-point Likert scale since adolescents reported difficulties with the 7-point scale.

**Table 3:** **Final TESQ-E questionnaire**

**Approach**	**Addressing the temptation directly**
*Strategy 1*	*Avoidance of temptations*
Item 1	If I am in town, I make sure that I don’t go by fast-food places
Item 2	If I pass a bakery, I avoid looking at display in the window
Item 3	If I go to the supermarket, I avoid the candy department
Item 4	If I am bored, I stay away from the kitchen
*Strategy 2*	*Controlling temptations*
Item 5	If I want to have a treat, I take a little bit and put the rest out of sight
Item 6	If I am watching TV, I make sure that the crisps are out of reach
Item 7	If I am behind the PC, I make sure there is some healthy food within reach
Item 8	If I want to eat candy, I take a few and put the rest of the bag away
**Approach**	**Addressing the psychological meaning of the temptation**
*Strategy 3*	*Distraction*
Item 9	If I feel tempted to buy candies, I distract myself
Item 10	If I feel like eating something, I call a friend instead
Item 11	If I am getting hungry before dinner, I try to keep myself busy
Item 12	If I have the urge to eat candy, I find something else to do
*Strategy 4*	*Suppression*
Item 13	If I pass a bakery, I ignore the smells of tasty foods
Item 14	If I want to eat unhealthy things, I just tell myself “no!”
Item 15	I use willpower to stay away from unhealthy snacks
Item 16	If I go to a party with lots of snacks, I ignore the food
**Approach**	**Addressing the goal directly**
*Strategy 5*	*Goal and rule setting*
Item 17	I plan to bring a piece of fruit to school
Item 18	I have an agreement with myself about how many candies I can have per day
Item 19	If I want to eat a snack, I take a piece of fruit first
Item 20	I set goals to eat healthily for myself
*Strategy 6*	*Goal deliberation*
Item 21	If I want to have a snack, I try to realize that snacks are bad for your health
Item 22	If I think I may be overeating, I think of how this may compromise exercising
Item 23	If I want to take a snack, I remember that I want to stay attractive
Item 24	If I feel like eating something unhealthy, I think about whether I really want it

Note: Answering options are 1 = never, 2 = sometimes, 3 = regularly, 4 = often, 5 = always.

The following specific instructions were given: “*Nowadays, tasty but often unhealthy food is available everywhere. Fast food can be bought at practically every street corner, in schools and at sport clubs. How do you deal with all this tasty food in your environment? Below you find a number of statements about dealing with food. Please circle the answer that best describes you. Think about what you did during the past two weeks when answering these questions. For example: Take the statement “If I have eaten something sweet, I brush my teeth”. If you have never done this in the past two weeks, circle the answer “never”. If you have often brushed your teeth after eating something sweet, you can circle the answer “often”.*

At all steps, items were formulated in English first. The researchers in the individual countries translated the English items to the national language. In each country, a person not involved in the project but fluent both in English and in the national language translated the items back to English. Adolescents in participating countries answered the questions in their country’s national language only. Hence, the TESQ-E is available in eight languages, i.e., English, Finnish, Danish, German, Polish, Portuguese, Romanian, and Dutch (this version was also used in Belgium)^b^.

The reliability and validity of the 24-item TESQ-E were subsequently determined in three separate data collections. All data collection procedures that involved adolescents were conducted in schools, complying with the ethical guidelines that specifically applied to the countries involved (i.e., when medical ethical approval was required, approval was established).

## Study 2 Validity and reliability of the TESQ-E

### Aim

The aims of study 2 were threefold. Firstly, study 2 aimed to evaluate the psychometric properties of the questionnaire. The hypothesized factor structure was tested to demonstrate that the 24 items describe six specific self-regulation strategies, and these six strategies describe three general self-regulation approaches, which can be grouped under one overarching concept of self-regulation. Next, internal reliabilities of the six scales were determined. Further, we aimed to assess the predictive validity of the TESQ-E scale, by assessing the correlations between the six self-regulation strategies and eating-related behaviors as well as dietary intake. A positive association is expected between self-regulatory strategy use and weight-related considerations, because being concerned about one’s weight and eating pattern, is considered a prerequisite for self-regulation of eating behavior. We expected a negative association between self-regulatory strategies, the extent to which snacking occurs habitual and the power of food (the influence the mere presence of food has). Individuals, who apply self-regulation strategies more often, are hypothesized to have weaker snacking habits and are less strongly influenced by the mere presence of food in their environment, as they will be better able to navigate the obesogenic food environment. Finally, we expect individuals with higher scores on the TESQ-E strategies to also report healthier dietary intakes.

### Method

#### Participants, design and procedure

Public schools in nine European countries (Belgium, Denmark, Finland, Germany, Poland, Portugal, Romania, The Netherlands, and the United Kingdom) were recruited for participation in a cross-sectional survey. Schools were selected that represented variety in rural and urban regions as well as high and low SES areas. Youngsters aged 10 to 17 were asked to complete the survey in one session in class. Completing the questionnaire took approximately 30 minutes. Schools were allowed to choose between computer-based or paper-and-pencil questionnaires. Of the total sample, 15.3% of the adolescents completed a computerized version of the questionnaire. The questionnaire assessed background characteristics, the TESQ-E, eating-related behavior (habit of snacking, weight-related considerations power of food, and power of food), and dietary intake (soft drink, fruit, vegetable, and snack consumption, and as well as breakfast frequency). A total of 121 schools participated, with 58.5% of these schools located in urban areas and 52.5% of these schools being situated in areas with a high socio-economic status. The questionnaire was completed by 11,392 adolescents.

#### Measures

TESQ-E assessed self-regulation strategies for healthy eating with 24 items as outlined in Table 3. Higher scores indicate more frequent use of self-regulation strategies.

Weight-related considerations were measured with three items derived from an existing scale [39]. Items are ‘I am concerned about being overweight’, ‘I pay attention to what I eat so I don’t gain weight’, and ‘My belly is too fat’ (range 1 = strongly disagree to 5 = strongly agree). Internal consistency was satisfactory (Cronbach’s alpha = .70) and a mean score was computed. Higher scores mean that participants are more concerned about their body weight.

Habit of snacking was assessed with six items from the self-reported habit index (SRHI) [40], comprising two core elements of habits (i.e., frequency and automaticity). Sample items are ‘unhealthy snacking is something I do frequently’ and ‘unhealthy snacking is something I do without thinking’ (1 = strongly disagree, 5 = strongly agree). Internal consistency was good (Cronbach’s alpha = .86) and a mean score was computed. A higher score indicates that participants have a stronger habit of snacking unhealthy foods.

The Power of Food scale (child/adolescent version) assesses the extent to which adolescents are influenced by the mere presence or availability of food [41]. An abbreviated version of eleven items (based on a selection approved by the authors of the scale; original 19-item version available from lowe@drexel.edu)^c^ was employed including, for example, “I think I enjoy eating a lot more than most other kids”, or ‘I think about food even when I’m not truly hungry (1 = strongly disagree, 5 = strongly agree). Internal consistency was good (Cronbach’s alpha = .86) and a mean score was computed. A higher score indicates that participants are influenced more strongly by the mere presence of food.

Dietary intake was assessed with four single items on the average daily intake of sugar-sweetened beverages, snacks, fruits and vegetables as prototypical (un)healthy foods that adolescents may (or may not) consume (cf. [17,42]. Specifically, adolescents were required to indicate their consumption on a 0 (less than 1/none) to 5 (more than 4) scale by the following items:

1. How many glasses of soft drinks, lemonade or energy drinks do you drink on an average day? (Don’t count light drinks and mineral water)
2. How many servings of fruit do you eat on an average day? (One serving is about one handful)
3. How many serving spoons of cooked or raw vegetables do you eat on an average day? (One serving spoon is about one handful)
4. How many snacks do you eat on an average day? (You may count the following as one snack: e.g., a handful of crisps, a candy bar, a sausage roll). The examples were country-specific, mentioning the most frequently consumed snacks and the reference size in that specific country).

The variables were analyzed independently. Higher scores indicate a higher consumption of that food.

Frequency of breakfast was assessed by asking on how many days per week, participants generally ate breakfast. Four answering options were given, 0 = never, 1 = once to three times a week, 2 = four to six times per week, 3 = seven days a week. Higher scores thus indicate a higher breakfast frequency.

Weight status: Weight and height were reported by the participants, from which BMI could be calculated. Because adolescents are still growing, adult BMI cut-offs cannot be used in adolescents and a categorization was made based on the widely used international (International Obesity Task Force) age and gender-specific cut offs for children and adolescents [43,44]. BMI was categorized into underweight, normal weight, overweight and obesity.

The Family Affluence Scale (FAS) was used as an indicator for socio-economic status (SES). The Family Affluence Scale [45] includes four items asking about family wealth. Items are 1) “Does your family own a car, van or truck?” (answering options: no; yes, one; yes, two or more, 2) “Do you have your own bedroom for you alone” (no, yes), 3) “During the past 12 months, how many times did you travel away on holiday with your family” (not at all; once; twice; more than twice), and 4) “How many computers does your family own?” (none; one; two; more than two). Following the procedure adopted in recent studies of the Health Behavior in School-aged Children (e.g., [45]), the two highest response categories were combined in items three and four. Three categories were then created based on the summed FAS score, indicating low affluence (FAS between 0 and 3), middle affluence (FAS score 4 or 5) and high affluence (FAS score 6 or 7).

Immigrant status was assessed by asking respondents what language they usually spoke with their parents (e.g., [46]). A dichotomous variable was computed for country’s national language (score = 0 and indicates a non-immigrant status) and other language (score = 1 and indicates an immigrant status).

### Results

#### Participants

Mean age was 13.21 (SD = 2.00) years. A total of 23.3% of the sample was 10 or 11 years old, 34.0% were 12 or 13 years old, 27.0% were 14 or 15 years old, and 15.8% were 16 or 17 years old. Of the sample, 50.5% were girls, and 90.7% spoke the country’s national language. Of the respondents, 13.0%, 39.3% and 47.8% were from low, middle or high affluent families. The majority of the sample had a normal weight (74.8%). A total of 10.5% were classified as underweight, 12.5% as overweight, and 2.1% as obese.

#### Structure of the TESQ-E

**Figure 1:**
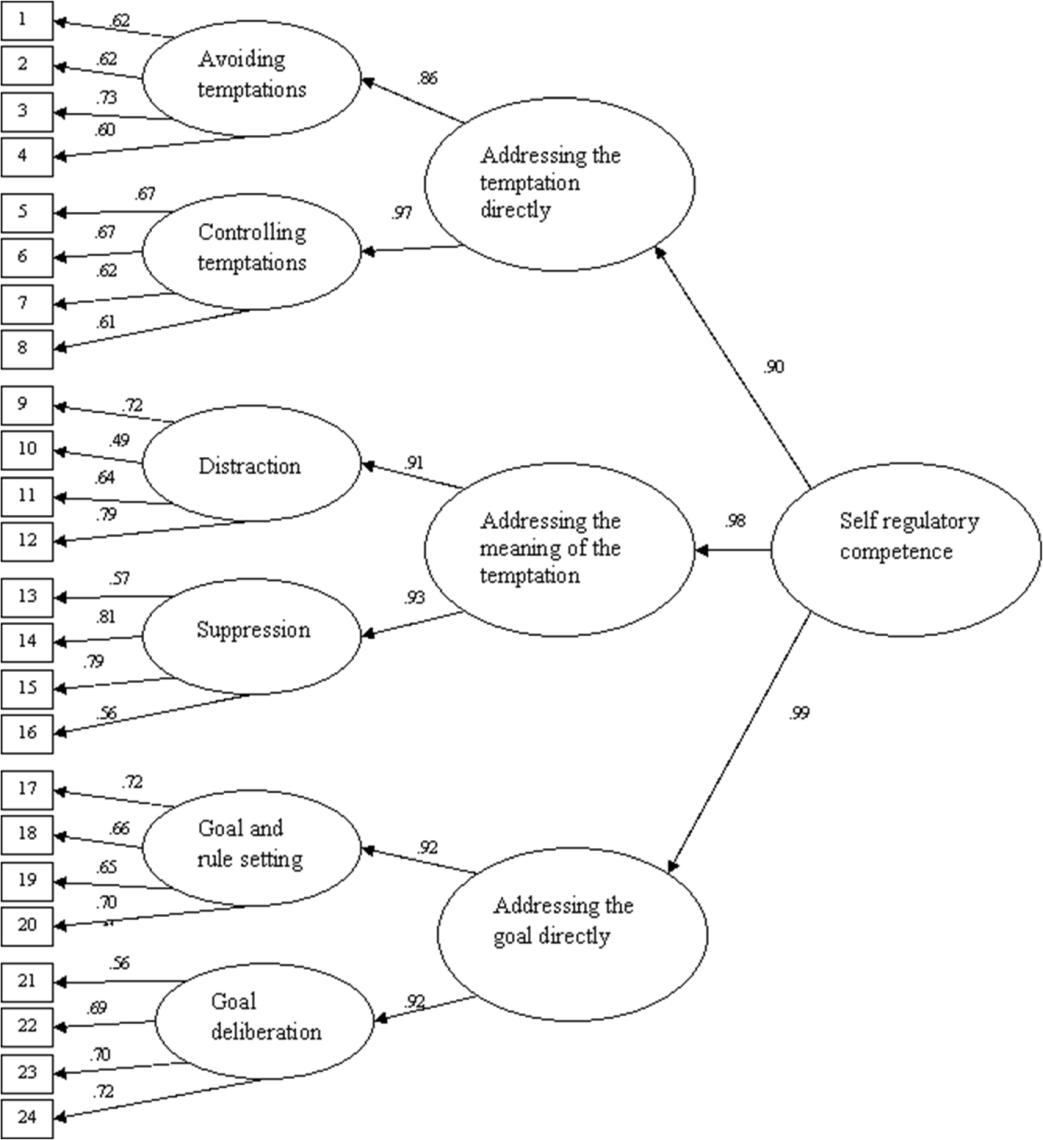
**Confirmatory factor analysis presenting standardized regression weights (n = 11,392).**

Confirmatory factor analysis (CFA) with expectation-maximization estimation in AMOS 17.0 was used in order to examine the theoretical model that six specific self-regulation strategies loaded on three general self-regulation approaches which are assumed to represent one higher-order factor, i.e., self-regulatory competence. To evaluate the model, absolute (root mean square error of approximation [RMSEA]) and incremental fit indices (Normed Fit Index [NFI] and Comparative Fit Index [CFI] were calculated. A good model fit is obtained when the NFI and CFI are higher than .95 and the RMSEA is less than .06 [47–49]. The model showed good fit (NFI = .95, CFI = .95, RMSEA = .04). The final model is graphically depicted in Figure 1.

#### Reliability

Cronbach’s α of the six strategies ranged from .73 to .78. Reliability did not improve if specific items were removed from the six subscales. The internal consistencies of the strategies were thus satisfactory and comparable across strategies. Internal consistencies for each of the strategies in the separate studies is reported in Table 4.

**Table 4:** **Means (M), standard deviations (SD) and correlations between the TESQ-E subscales (Study 2,** ***n =*** **11,392)**

	**1**	**2**	**3**	**4**	**5**	**6**	**7**	**8**	**9**	**10**
Avoidance (1)	-	.62*	.55	.60	.49	.53	.90	.63	.56	.77
Controlling temptations (2)			.59	.60	.61	.60	.90	.65	.67	.82
Distraction (3)				.68	.61	.63	.63	.92	.69	.82
Suppression (4)					.64	.64	.67	.92	.70	.84
Goal and rule setting (5)						.63	.61	.68	.90	.82
Goal deliberation (6)							.63	.69	.90	.83
Addressing temptations directly (7)								.71	.69	.88
Addressing meaning of temptation (8)									.76	.91
Addressing the goal directly (9)										.91
Self regulatory competence (10)										
M	2.24	2.49	2.36	2.25	2.44	2.57	2.36	2.30	2.50	2.39
SD	0.96	0.97	0.93	0.93	1.02	1.03	0.87	0.85	0.93	0.80
Cronbach’s alpha study 2	.73	.74	.75	.77	.76	.78	.83	.85	.85	.93
Cronbach’s alpha study 3	.63	.63	.78	.75	.73	.70	.75	.86	.82	.92
Cronbach’s alpha study 4	.74	.75	.69	.78	.78	.84	.83	.83	.89	.94

Note. *All *p’*s < .001.

#### Descriptive information and correlations between the TESQ-E subscales

Because the hypothesized factor structure was confirmed and the reliability was satisfying, six TESQ-E subscales were established by computing the mean of the four items of each subscale. Table 4 presents the means, standard deviations, and correlations of the TESQ-E subscales. All TESQ-E strategies correlated moderately to strongly.

### Predictive validity

To determine whether the TESQ-E is associated with eating-related behaviors and dietary intake, bivariate Pearson correlations between the TESQ-E subscales and weight-related considerations, snacking habits, power of food, and dietary intake (soft drink, fruit, vegetable, snack consumption and breakfast frequency) were computed (Table 5). Adolescents who reported to use self-regulation strategies more frequently, also generally reported weaker habits of snacking, more weight-related considerations, lower power of food, lower intake of soft drinks and snacks, higher fruit and vegetable intake and breakfast frequency.

**Table 5:** **Correlations between TESQ-E subscales and eating-related behaviors and dietary intake (Study 2, n = 11,392)**

**TESQ-E**	**Weight-related considerations**	**Snacking habits**	**Power of food**	**Soft drink intake**	**Fruit intake**	**Vegetable intake**	**Snack intake**	**Breakfast frequency**
Avoidance	.24	-.24	-.13	-.20	.18	.13	-.32	.11
Controlling temptations	.26	-.30	-.16	-.22	.24	.20	-.32	.15
Distraction	.33	-.18	-.07	-.19	.21	.16	-.29	.09
Suppression	.33	-.24	-.12	-.20	.23	.15	-.31	.07
Goal and rule setting	.31	-.26	-.08	-.22	.38	.23	-.25	.12
Goal deliberation	.41	-.19	-.06	-.20	.23	.16	-.26	.08
Addressing temptations directly	.28	-.30	-.16	-.23	.23	.18	-.36	.14
Addressing meaning temptation	.36	-.23	-.10	-.21	.24	.17	-.33	.08
Addressing the goal directly	.40	-.25	-.07	-.24	.34	.21	-.28	.11
Self regulatory competence	.39	-.29	-.12	-.25	.30	.21	-.36	.13

Note. All *p’s* < .001.

## Study 3 Test-retest reliability

### Aim

The aim of the third study was to assess test-retest reliabilities of the six self-regulation strategies of the TESQ-E scale over a four-week period. Because the TESQ-E proved to have good psychometric qualities, test-retest reliability was assessed in one European country. We expected good test-retest reliabilities. It is generally suggested that test-retest reliability is good when the correlations between assessments over a two-week period exceed .70.

### Method

#### Participants, design and procedure

In order to examine the test-retest reliability of the TESQ-E, a sample of 140 Romanian adolescents (mean age = 13.29, SD = 2.37; 57.1% girls) were asked to complete a survey assessing the TESQ-E twice four weeks apart.

#### Measures

The TESQ-E was assessed at baseline and again four weeks later. Mean scores were computed for the six separate strategies, with higher scores indicating more use of self-regulation strategies for healthy eating.

### Results

The correlations between baseline and four weeks later were r = .55 and r = .57 for avoiding and controlling temptations, respectively. Correlations between the two assessments were .66 and .71 for distraction and suppression, respectively. For goal setting and goal deliberation, test-retest reliabilities were r =. 74 and r = .71 respectively. For the three higher-order self-regulation approaches, correlations were r = .63, r = .76, and r = .79 for strategies addressing temptations directly, for strategies addressing the meaning of temptations and for strategies addressing the goal directly, respectively. The test-retest reliability for the complete self-regulatory competence scale was .81. Thus, test-retest reliabilities were moderate for strategies related to addressing temptations directly, and good for strategies addressing the psychological meaning of the temptations, and strategies addressing the goal directly, as well as for the overall scale.

## Study 4 Construct validity

### Aim

The aim of the fourth study was to establish construct validity of the TESQ-E strategies, by correlating the strategies with general self-regulation measures (trait self-control, delay of gratification, adolescent self-regulation), and related concepts such as motivation and autonomy. We expected positive, but only modest correlations with all three general self-regulation measures. Although being generally capable of self-regulation facilitates the use of specific self-regulatory strategies, it does not necessarily mean that a general ability to self-regulate is translated into the use of specific strategies for healthy eating.

Compared to the general self-regulation measures, we expected stronger positive correlations between the TESQ-E strategies and motivation. Motivation is an important predictor of self-regulation. If individuals are not motivated to eat healthily, there will be no need to use self-regulation strategies. However, motivation is not an equivalent of self-regulation and in that sense reflects a theoretically distinct construct.

Agentic or ‘good’ autonomy reflects autonomy driven by an authentic wish to gain agency and to learn to responsibly regulate oneself. Self-presentational or ‘bad’ autonomy reflects autonomy driven by a desire to break away from the familial ties and to show off this new-found independence to peers [10]. Based on research showing that agentic autonomy correlated positively and self-presentational autonomy correlated negatively with unhealthy snack purchase [10], we expected that the TESQ-E correlated positively with agentic autonomy, and negatively with self-presentational autonomy.

### Method

#### Participants, design and procedure

One public school participated with four classes at varying educational levels. A total of 93 Dutch adolescents completed a questionnaire assessing the TESQ-E, self-control, delay of gratification, short and long-term self-regulation, autonomy, and motivation. Mean age was 14.76 (SD = .69) and 68.8% were girls.

#### Measures

The TESQ-E was assessed as outlined in Table 3. Mean scores were computed for the six strategies, separately.

Trait self-control was assessed with the brief version of the Self-Control Scale [50]. This scale consists of 13 items on self-control, including such items as “I find it hard to quit bad habits” (reversed) and “I wish I had more discipline” using a 5-point Likert scale (ranging from 1 = not at all true for me to 5 = totally true for me). Cronbach’s alpha was .78 and a mean score was computed. Higher scores mean more self-control.

Delay of gratification (i.e., general ability to resist a smaller, but immediate, reward in order to obtain a larger, but delayed reward [51]) was assessed by presenting individuals with a (hypothetical) choice about a monetary reward in exchange for their participation in the study, either a small immediate reward (7 €) or a larger delayed reward (10 € one week later) (cf. [51]). A higher score means that participants are better able to delay gratification.

The Adolescent Self-Regulatory Inventory (ASRI) [52] is a 27-item questionnaire specifically designed for adolescents and assesses short-term (15 items) and long-term self-regulation (12 items). Items are measured on a 5-point Likert scale (ranging from 1 = not at all true for me to 5 = totally true for me). Sample items include “After I’m interrupted or distracted, I can easily continue working where I left off”(short-term) and “I can find ways to make myself study even when my friends want to go out” (long-term). Cronbach’s alpha was .74 and .79 for short-term and long-term self-regulation respectively. Higher scores mean better self-regulation.

Autonomous and controlled motivation for healthy eating was assessed with 15 items based on the Regulation of Eating Behaviors Scale [53]. The scale assesses motivational orientations toward dietary regulation. Participants are asked what specific reason to eat healthily applies to them personally on a 7-point Likert scale (1 = not at all true to 7 = very true). The autonomous motivation subscale includes nine items (e.g., “Eating healthy is part of the way I want to live my life”). The controlled motivation subscale (e.g., “Other people close to me insist that I eat healthy) includes six items. Cronbach’s alpha’s were .91 and .75 for autonomous and controlled motivation respectively and mean scores were computed. Higher scores indicate stronger autonomous and controlled motivations.

Agentic autonomy was assessed by five items from the behavioral autonomy subscale of the Worthington Autonomy Scale [54]. Items were measured on a 5-point Likert scale (ranging from 1 = totally disagree to 5 = totally agree). Sample items include “I accept responsibility for my own mistakes” and “I don’t spend my money wisely (reverse coded). Cronbach’s alpha was .36, which is lower than previously found for this measure. A mean score was computed. A higher score indicates more agentic autonomy.

Self-presentational autonomy was assessed by asking adolescents to indicate to what extent they agreed (1 = totally disagree to 5 = totally agree) with five statements, such as “I like to show others that I don’t mind to take risks” and “I want to be seen as someone who makes his own decisions”. Also, for self-presentational autonomy, internal consistency was low (Cronbach’s alpha = .46). A mean score was computed. Higher scores indicate more self-presentational autonomy.

### Results

Table 6 presents the correlations of TESQ-E strategies with related concepts of self-regulation.

**Table 6:** **Correlations between TESQ-E subscales and general indicators of self-regulation (Study 4, n = 93)**

**TESQ-E**	**Self-control**		**Delay of gratification** ^**a**^		**Short-term self-regulation**		**Long-term self-regulation**		**Autonomous motivation**		**Controlled motivation**		**Agentic autonomy**		**Self-presentational autonomy**	
	***r***	***p***	***r***	***p***	***r***	***p***	***r***	***p***	***r***	***p***	***r***	***p***	***r***	***p***	***r***	***p***
Avoidance	.09	.41	.15	.15	.09	.42	.13	.22	.34	.001	.43	<.001	.27	.009	-.21	.047
Controlling temptations	.30	.004	.13	.22	.20	.06	.27	.01	.45	<.001	.39	<.001	.28	.008	-.12	.26
Distraction	.21	.047	-.01	.93	.11	.29	.29	.005	.43	<.001	.46	<.001	.34	.001	.02	.82
Suppression	.09	.42	.14	.19	-.04	.72	.07	.53	.53	<.001	.58	<.001	.34	.001	-.02	.84
Goal setting	.17	.10	.25	.02	.12	.26	.25	.02	.67	<.001	.53	<.001	.35	.001	-.02	.84
Goal deliberation	.18	.08	.21	.05	.02	.83	.16	.12	.58	<.001	.55	<.001	.35	.001	-.14	.19
Addressing temptations directly	.21	.04	.15	.16	.16	.13	.22	.03	.44	<.001	.46	<.001	.31	.003	-.19	.08
Addressing meaning temptation	.16	.13	.07	.54	.04	.74	.19	.07	.54	<.001	.58	<.001	.37	<.001	.000	.996
Addressing the goal directly	.19	.07	.24	.02	.07	.49	.22	.04	.67	<.001	.58	<.001	.38	<.001	-.09	.40
Self regulatory competence	.21	.048	.18	.09	.10	.36	.23	.03	.61	<.001	.60	<.001	.39	<.001	-.10	.34

^a^Because delay of gratification is a dichotomous measure, these correlations reflect Spearman’s rho. All other correlations are Pearson correlations.

- **Self-control:** Controlling temptations and distraction were significantly related to trait self-control. Adolescents high in trait self-control reported to use these strategies more often. Avoidance, suppression, goal setting and goal deliberation were not significantly related to trait self-control. Two of the three approaches (addressing temptations directly and addressing the meaning of temptations) as well as overall self-regulatory competence were also positively associated with trait self-control.
- **Delay of gratification:** Adolescents, who reported to apply goal setting and goal deliberation frequently, were significantly better able to delay gratification. Avoidance, controlling temptations, distraction and suppression were unrelated to delay of gratification. With respect to the self-regulation approaches and general self-regulatory competence, only strategies addressing the goal directly were significantly associated with delay of gratification.
- **Short- and long-term self-regulation:** None of the specific strategies, the self-regulation approaches or general self-regulatory competence were significantly related to short-term self-regulation. Controlling temptations, distraction and goal setting were significantly positively related to long-term self-regulation, whereas the other three strategies were unrelated to long-term self-regulation. Two of the three self-regulation approaches (addressing temptations and goal directly) and general self-regulatory competence were significantly related to long-term self-regulation.
- **Motivation for eating healthily:** All six specific strategies, the three self-regulation approaches and general self-regulatory competence correlated moderately to strongly positively with both autonomous and controlled motivations.
- **Autonomy:** All six strategies, three self-regulation approaches and general self-regulatory competence were significantly positively related to agentic autonomy. Only avoidance of temptations correlated significantly and negatively with self-presentational autonomy. This indicates that individuals report to use self-regulatory strategies more often may do so for reasons of expressing agency than for reasons of showing-off independence.

## General discussion

The present research aimed to identify theory-based self-regulation strategies adolescents report to use and to develop and validate a questionnaire to assess these strategies. In study 1, adolescents themselves described their ways of self-regulating foods in their own words, thereby ensuring that the types of strategies and how they were named were relevant to adolescents and contributing to the ecological validity of the scale. Next, input from the adolescents was categorized into strategies and strategies into approaches that were derived from a theoretical framework distinguishing between temptation-directed and goal-directed strategies. This resulted in the 24-item TESQ-E questionnaire assessing six strategies. The hypothesized factor structure was confirmed in study 2. Studies 2 to 4 further showed that the self-regulation strategies were relevant correlates for a variety of eating-related behaviors and dietary intake. The questionnaire demonstrated acceptable test-retest reliabilities and correlated moderately with related concepts suggesting that the scale is distinguishable from available instruments of general self-regulation, motivation and autonomy. To our knowledge, this is the first time that such a theoretical distinction has been put to an empirical test and proven valid in a large and diverse sample of young people. The six strategies that resulted from our analyses in study 1 have proven to represent meaningful distinct strategies, although they were interrelated. Notably, we did not find evidence for the existence of strategies relating to altering the psychological meaning of a goal, as proposed by the theoretical framework by Fishbach and Converse [29]. The absence of this fourth category of strategies may have different causes and at this point we can only speculate about the exact reason. One reason may be that our research involved young people for whom altering the psychological meaning of a personal goal may be too advanced. None of the adolescents participating in our study did spontaneously mention behaviors that could be considered strategies to alter the meaning of a goal. This may be because adolescents do not consider such actions as strategies they can apply in dealing with foods, or never use these strategies because their ability of future oriented thinking is limited compared with adults. Future research should address this issue in sample involving adults as well.

The six strategies further proved to be associated with eating-related behaviors and dietary intake in a meaningful way. Self-regulation strategies were associated with weight-related considerations, such as paying attention to food intake to prevent overweight. It is unclear whether such concerns result from engaging in self-regulation, are a prerequisite for engaging in self-regulation, or simply are an expression of self-regulation. The adoption of self-regulation strategies was also associated with a weaker habit of snacking, though the magnitude of the correlations varied across strategies. This may suggest that self-regulation strategies could contribute to counteracting the mindless automatic and frequent consumption of snacks [22]. We observed weak associations of self-regulation strategies with the Power of Food scale [41], suggesting that the adoption of strategies is not dependent on the extent that adolescents are aware of and sensitive to their obesogenic food environment. With respect to self-reported intake of foods, the strongest correlations were observed for snack intake: adolescents who reported to engage in self-regulation strategies were better able to refrain from snack consumption. This is relevant because snack consumption is known to be an important contributor to overweight, especially in adolescents [5,55]. Self-regulation strategies were also associated with two other eating behaviors, i.e., less consumption of soft drinks and higher consumption of fruits, but only weakly with vegetable consumption and breakfast consumption. The latter two behaviors may be more under parental control, and therefore less susceptible to self-regulation by adolescents themselves.

Study 3 showed that test-retest reliabilities for the six strategies over a four week period were satisfactory. The four week period was longer than the generally used two week reference period for evaluating test-retest reliabilities. Because the questionnaires were administered in schools, schools’ schedules were leading in timing the questionnaires and a two week time frame was not considered feasible. Consequently the test-reliabilities may have been slightly lower.

Study 4 indicated that the six TESQ-E strategies showed a meaningful pattern of associations with related concepts of self-regulation. Controlling temptations and distraction correlated with dispositional self-control, demonstrating that the engagement in these strategies relates to the ability to interrupt or overrule undesired response tendencies [50]. The fact that the other strategies were not associated with self-control may suggest that employing these strategies does not depend on dispositional self-control even though they involve active self-regulation. Only strategies relating to goal-directed strategies, such as goal setting and goal deliberation, were associated with delay of gratification. In order to be able to delay gratification, one needs to activate a future mindset [51]. The ability to think about the future and to adopt a long-term perspective is also required to set goals and deliberate on these goals, and may therefore explain why specifically these strategies were associated with delay of gratification. Remarkably, none of the TESQ-E strategies were associated with short-term, but temptation- and goal-directed strategies were associated with long-term regulation as assessed by the Adolescent Self-Regulatory Inventory [52]. The employment of any of the six strategies was clearly associated with (either controlled or autonomous) motivation and with agentic autonomy, but not with autonomy for self-presentation reasons. This may suggest that the source of motivation does not matter so much for self-regulation as long as individuals are motivated, but that reasons for being autonomous do affect engagement in self-regulation (cf., [56]). Yet, it should also be acknowledged that the measures for autonomy generally had a low reliability. It might be that the validity and reliability of these measures is not sufficient for 10 to 17 year olds, although it should be noted that a previous study among 14 to 17 year old adolescents found more satisfying reliability estimates for the autonomy measures [10].

Several limitations of the studies need to be acknowledged. The most important limitation is that all studies relied on self-report measures. For example, for height and weight, self-report may be unreliable because adolescents do not exactly know their weight. But also self-reported dietary intake and use of self-regulation strategies may be subject to biases, because individuals may experience difficulties in recalling and accurately assessing their behavior. A further disadvantage is that self-report measures may be vulnerable to socially desirable answering. However, the topics of the questionnaire were not very emotionally charged and the questionnaire was completed anonymously and in private. Another limitation is the use of cross-sectional designs. Therefore, predictive validity was not fully established as that would require longitudinal data. Further, although data were collected from different classes across studies, a few schools were included in more than one study. Because there might be higher resemblance between participants from the same school, the questionnaire validation across studies might show better results compared to when completely independent samples from different schools had been used in the different studies**.** Finally, it should be mentioned that a small proportion of adolescents in study 2 completed a computerized version of the questionnaire. We have not compared psychometric properties of the computerized and paper-and-pencil versions of the questionnaires. However, based on two meta-analyses that showed that both modes of data collection yield largely similar results [57,58], this is deemed not too problematic.

Our research has a number of advantages. First, our research combines a theoretically driven approach of distinguishing several types of self-regulation strategies with a bottom-up approach of statements generated by adolescents themselves, which contributes to the validity of the scale in this particular group of adolescents. Second, we employed a large, culturally diverse and representative sample of European young people that allows for a thorough investigation of the self-regulation strategies that adolescents employ to regulate their eating behavior. In addition, good cross-cultural validity was demonstrated with secondary analyses of the TESQ-E that are reported elsewhere (internal Tempest report) [Sørensen B, Nureeva L, Brunsø K, Baban A, Gaspar T, De Ridder DTD: Cross-Cultural Validity of the TESQ-E Scale in Nine European Countries. Submitted for publication]. Nevertheless, the scale was developed and validated in Europe, and the questionnaire needs to be validated first before it can be used reliably outside Europe.

## Conclusions

The TESQ-E assesses six self-regulation strategies that adolescents can use to eat healthily. To broaden the scope of the instrument, future research should investigate whether the TESQ-E can be translated to other health behaviors (e.g., alcohol intake, exercise) as well as to other populations (e.g., adults) and cultures (e.g., US teens).

The TESQ-E is an instrument that can be used by researchers who are interested in the processes explaining why adolescents do eat (un)healthy as well as by practitioners who aim to design or evaluate interventions to improve the use of self-regulation strategies to ensure healthy eating. Depending on the purpose of using the TESQ-E, the six specific strategies can be used separately, be combined into the three categories or into a more crude measure for general self-regulation for healthy eating consisting of all 24 items. For instance, one could use the six specific strategies of the TESQ-E to identify intervention targets (e.g., to develop an intervention that targets specific strategies because adolescents do not frequently use them). In addition, the TESQ-E can be used for the evaluation of interventions to assess whether self-regulation has been improved as a result of intervention. For this purpose one could use the three self-regulation approaches. These three approaches are also relevant if one is investigating the relation between self-regulation and actual eating-related behavior and dietary intake. The theoretical ground of these approaches allows for the formation of specific hypotheses about how and under what conditions self-regulation supports healthy eating. For example, it can be expected that especially in food-rich environments using temptation-directed strategies are relevant [59]. Finally, in a more practical way, the questionnaire may also be used by parents and other caregivers to gain insight into the self-regulation strategies that adolescents could use to regulate their dietary intake.

## Endnotes

^a^Tempest Internal reports are available upon request from the authors.

^b^The TESQ-E questionnaire assesses the self-reported use of six self-regulation strategies. Throughout the rest of the manuscript, mentioning of use of self-regulation strategies refers to the self-reported use. Please also note that the TESQ-E is freely available in eight languages from the TEMPEST project website: www.tempestproject.eu.

^c^The eight items that were excluded from the original Children’s Power of Food Scale were items 2, 5, 8, 9, 1, 12, 14 and 16.

## Abbreviations

TESQ-E: Tempest self-regulation questionnaire for eating
ASRI: Adolescent self-regulation inventory
